# Mechanisms of Neuronal Death in the Cerebral Cortex during Aging and Development of Alzheimer’s Disease-Like Pathology in Rats

**DOI:** 10.3390/ijms20225632

**Published:** 2019-11-11

**Authors:** Darya V. Telegina, Gleb K. Suvorov, Oyuna S. Kozhevnikova, Nataliya G. Kolosova

**Affiliations:** 1 Institute of Cytology and Genetics, Siberian Branch of Russian Academy of Sciences, Pr. Lavrentyeva 10, Novosibirsk 630090, Russia; telegina@bionet.nsc.ru (D.V.T.); augustocash@gmail.com (G.K.S.); oidopova@bionet.nsc.ru (O.S.K.); 2 Novosibirsk State University, 1 Pirogova str., Novosibirsk 630090, Russia

**Keywords:** Alzheimer’s disease, neuronal cell death, apoptosis, autophagy, necroptosis, OXYS rats

## Abstract

Alzheimer’s disease (AD) is the commonest type of late-life dementia and damages the cerebral cortex, a vulnerable brain region implicated in memory, emotion, cognition, and decision-making behavior. AD is characterized by progressive neuronal loss, but the mechanisms of cell death at different stages of the disease remain unknown. Here, by means of OXYS rats as an appropriate model of the most common (sporadic) AD form, we studied the main pathways of cell death during development of AD-like pathology, including the preclinical stage. We found that apoptosis is activated at the pre-symptomatic stage (age 20 days) correlating with the retardation of brain development in the OXYS strain early in life. Progression of the AD-like pathology was accompanied by activation of apoptosis and necroptosis resulting from a decline of autophagy-mediated proteostasis. Our results are consistent with the idea that the nature of changes in the pathways of apoptosis, autophagy, and necrosis depends on the stage of AD.

## 1. Introduction

Alzheimer’s disease (AD) is the commonest type of age-related dementia and shows increasing prevalence because of aging of the population [1]. AD is characterized by plaques of β-amyloid peptide and neurofibrillary tangles in the brain. Pathological protein aggregates formation impose stress onto neurons and cause cytotoxic events, which include increased production of reactive oxygen species, synaptic dysfunction, excitotoxicity, endoplasmic-reticulum stress, impaired protein degradation systems, mitochondrial dysfunction, DNA damage, inflammation, as well as cell cycle re-entry [2,3]. These are substantial neuronal challenges, and their pathological alterations eventually cause neurons to die. The clinical signs of AD are thought to correlate with the degree of cerebral neuronal loss that takes place over years and decades, particularly in the hippocampus and cerebral neocortex. Nonetheless, as noted in a recent review by Fricker et al., despite decades of research, we still have a poor understanding of how neurons die in AD and consequently only a limited ability to prevent this neuronal death [4]. Currently, AD still lacks an etiology, early prediction, diagnosis, and effective treatment [5].

Cell death is a highly organized basic activity that is as complex in terms of regulation as are cell division and differentiation. Currently, multiple forms of cell death are known, among which apoptosis, necrosis, and autophagy are the three main types [4]. Autophagy and apoptosis are major physiological processes that contribute to cellular homeostasis including neuronal one. Autophagy seems to be a major housekeeping mechanism that facilitates recycling of cell materials, utilizing them for the production of energy during emergencies. Autophagy involves processes targeting long-lived cytoplasmic proteins and impaired organelles and eventually delivers this targeted material into lysosomes. Autophagy is currently recognized as a master arbiter of decisions on neuronal death and survival in AD [6].

Apoptotic neuronal death features membrane blebbing, cell rounding, cytoskeleton breakdown, pyknosis of the nucleus, cytoplasm condensation and fragmentation, chromatin fragmentation and condensation, and generation of apoptotic bodies, which are rapidly phagocytosed by neighboring cells or macrophages [7]. Necroptosis is a form of regulated necrotic cell death that can be activated under apoptosis-deficient conditions [8]. Necroptosis can be triggered by the activation of death receptors (DRs) such as TNF receptor 1 (TNFR1). DRs activation causes the triggering of receptor-interacting protein kinase 1 (RIPK1) and RIPK1-kinase activity-dependent formation of an RIPK1–RIPK3 mixed-lineage kinase domain-like protein (MLKL) complex [8]. MLKL phosphorylation by RIPK3 ultimately results in necroptosis through disruption of the outer cell membrane and cell lysis [4,8,9].

Investigation of the cell death pathway during the development of AD is the biggest challenge for researchers owing to the lack of relevant biological samples at different stages of the disease. Postmortem samples of brain tissues can be used only to examine the end results of neurodegenerative processes, which do not necessarily reflect the mechanisms responsible for disease development. Out of the 66 existing transgenic mouse models incorporating familial amyloid precursor protein (APP) mutations with or without presenilin mutations (see http://www.alzforum.org/research-models), only seven manifest neuronal loss [4]. In the present study, we used non-transgenic senescence-accelerated OXYS rats, which spontaneously develop a phenotype similar to human AD and other age-related diseases [10,11,12,13,14]. OXYS rats reproduce all the major neuropathological features of AD although there are no mutations characteristic of familial AD in the genome of these rats [15]. Behavioral changes and memory and learning deficits in OXYS rats develop by age 3–5 months simultaneously with the first signs of neurodegeneration: Disruption of synapses, death of neurons, dysfunction of mitochondria, and hyperphosphorylation of the tau protein in the cerebral cortex and hippocampus. In the OXYS strain, with age, neurodegenerative alterations increase, accompanied by excessive production of APP and accumulation of β-amyloid peptide (Aβ) by age 12 months and well-pronounced amyloid-related pathology by age 18 months [12,15].

Previously, we have demonstrated impairment of the cell death pathway in OXYS rat’s retina during development of retinopathy [16,17]. Moreover, analysis of RNA sequencing data has revealed that gene expression changes during the development of AD-like pathology in the cortex of OXYS rats are related to cell death among other processes [18]. We hypothesized here that dysregulation of the cell death cascade participates in the neuronal degeneration in OXYS rats. Therefore, our aim was to assess the age-associated changes in autophagy, apoptosis, and necrosis in the cerebral cortex of OXYS and control (Wistar) rats at the ages of 20 days, five, and 18 months and contributions of cellular death to development of the AD-like pathology in OXYS rats.

## 2. Results

### 2.1. Evaluation of Apoptosis in the Cerebral Cortex of OXYS and Wistar Rats

**Figure 1:**
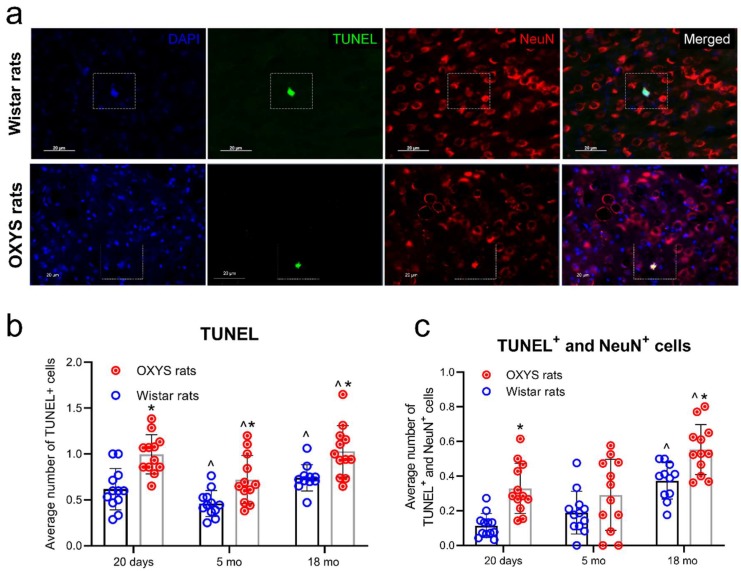
Apoptotic cell death. (**a**) Representative images of cortex cryosections (immunostained by the terminal deoxynucleotidyl transferase dUTP nick end labeling (TUNEL) method) from 18-month-old OXYS and Wistar rats. The TUNEL staining (green) and neuronal nuclear antigen (NeuN) staining (red) were examined under a fluorescence microscope, with subsequent quantitative analysis. The numbers of apoptotic cells (**b**) and apoptotic neurons (**c**) were calculated by averaging the numbers of TUNEL signals. Data are presented as mean ± SD, *n* = 4 (with three sections per animal). * Interstrain differences, ^ age-associated differences; *p* < 0.05.

The terminal deoxynucleotidyl transferase dUTP nick end labeling (TUNEL) assay was performed for the detection of apoptotic cells. Simultaneous immunostaining of NeuN (neuronal nuclear antigen) and TUNEL staining were applied for the detection of apoptotic neurons (Figure 1a).

At the age of 20 days, the numbers of both TUNEL + total cells and TUNEL + NeuN + neurons were greater (*p* < 0.05) in OXYS rats than in Wistar rats. By the age of five months, the numbers of apoptotic total cells and apoptotic neurons decreased in the cortex of OXYS and Wistar rats, but the number of TUNEL + total cells remained greater in OXYS rats. By age 18 months, the numbers of apoptotic total cells and apoptotic neurons increased in both strains as compared to age five months. Moreover, the numbers of TUNEL + total cells and TUNEL + NeuN+ neurons were greater in 18-month-old OXYS rats than in age-matched Wistar rats (Figure 1b,c).

### 2.2. Age-Related Alterations of Autophagy in the Rat Cerebral Cortex

ATG7 and ATG12 are required components of the autophagosome maturation pathway. Western blot analysis was performed to determine the age-related changes in their expression in the cortex among 20-day-old and five- and 18-month-old OXYS and Wistar rats. 

**Figure 2:**
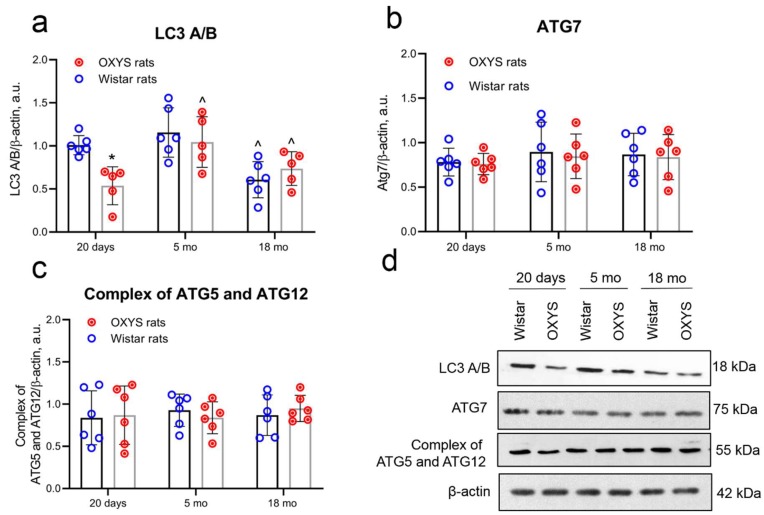
Alteration of autophagy proteins during aging and development of Alzheimer’s disease-like pathology. Protein expression of LC3 A/B (**a**), ATG7 (**b**), and of the ATG5–ATG12 conjugate complex (**c**) in the cortex from normal Wistar rats and from OXYS rats with Alzheimer’s disease (AD)-like pathology. (**d**) Representative immunoblots of LC3 A/B, ATG7, and of the ATG5–ATG12 conjugate complex in the cerebral cortex of OXYS and Wistar rats. Data are presented as mean ± SD, *n* = 5–6. * Interstrain differences, ^ age-associated differences; *p* < 0.05.

The western blotting revealed that the protein amounts of ATG7 and of the ATG5–ATG12 conjugated complex were not affected by either age or genotype. We detected no significant difference in ATG7 and ATG12 protein levels between Wistar and OXYS rats at the different ages (Figure 2b–d).

We measured LC3 A/B levels by western blotting and immunohistochemistry in the cortex from 20-day-old and five- and 18-month-old OXYS and Wistar rats. The LC3 A/B amount in the cortex of 20-day-old OXYS rats was lower compared to Wistar rats, as was shown by western blotting (*p* < 0.05). At the age of five months, the expression of LC3 A/B increased in OXYS rats (*p* < 0.05), while in Wistar rats, it did not change with age. No difference was noted between OXYS and Wistar rats at this age. The LC3 A/B levels in 18-month-old OXYS and Wistar rats (*p* < 0.05) were decreased compared to the age of five months (Figure 2a,d).

**Figure 3:**
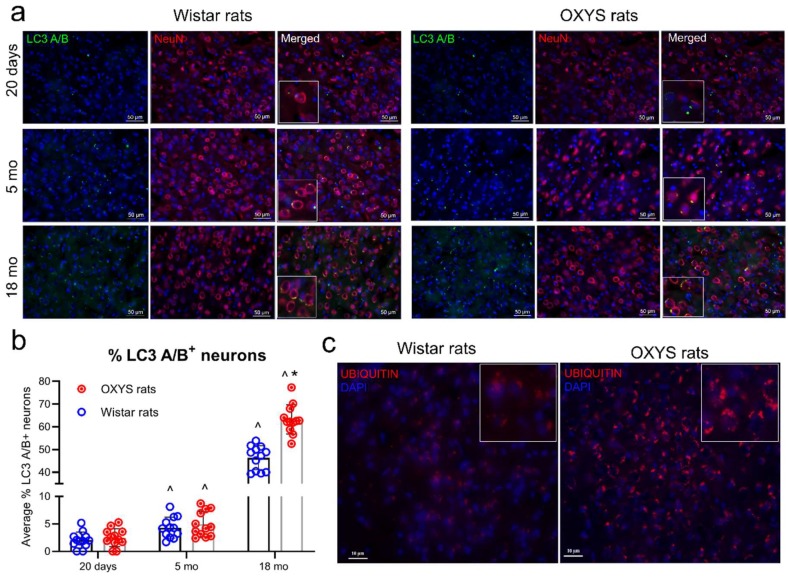
LC3 A/B expression in the Wistar and OXYS rats cortex at different ages. (**a**) Representative images of cortex cryosections immunostained for LC3 A/B (green) and the colocalization of LC3 A/B fluorescent dots with a marker of neurons (NeuN; red) in OXYS and Wistar rats. (**b**) Quantitative analysis yielded an increasing number of LC3 A/B^+^ neurons with age in both rat strains. (**c**) Immunofluorescence of ubiquitin signals was greater in the cerebral cortex of 18-month-old OXYS rats than in age-matched Wistar rats. Data are presented as mean ± SD, *n* = 4 (with three sections per animal). * Interstrain differences, ^age-associated differences; *p* < 0.05.

At the next step, we determined the number of neurons containing the LC3 A/B protein (LC3 A/B+ neurons) in the rat cortex by fluorescence microscopy (Figure 3a).

LC3 A/B labeling appeared as numerous fluorescent dots with strong immunoreactivity in the somas of some neurons. Our data suggested that the number of LC3 A/B^+^ neurons increased in both rat strains with age. At the age of 18 months, the number of LC3 A/B^+^ neurons significantly increased in the cortex of OXYS and Wistar rats. At this age, the number of LC3 A/B^+^ neurons was greater in OXYS rats than in Wistar rats (*p* < 0.05; Figure 3c). In accordance with these data, the levels of ubiquitinated proteins in the cerebral cortex of 18-month-old OXYS rats were significantly higher than those in age-matched Wistar rats, indicating impaired autophagy in OXYS rats (Figure 3b).

### 2.3. Localization of Necrosome Subunits RIPK1 and RIPK3 in the Rat Cortex

**Figure 4:**
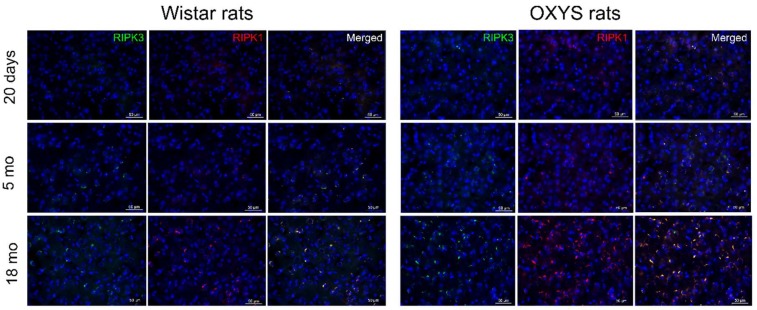
Markers of necroptosis. Representative images of cortex cryosections immunostained for receptor-interacting protein kinase 1 (RIPK1) (red) and RIPK3 (green) from 20-day-old and five- and 18-month-old OXYS and Wistar rats.

Necrosis is known to be a regulated process mediated by receptor-interacting protein kinases (RIPKs) [4,19]. The localization of proteins RIPK1 and RIPK3 in the cortex was determined by immunohistochemistry. RIPK1- and RIPK3-specific immunostaining was present in the brain sections in OXYS and Wistar rats of all ages. In the cortex of OXYS rats, the colocalization of RIPK1 and RIPK3 significantly increased only at the age of 18 months relative to Wistar rats (Figure 4).

### 2.4. Propidium Iodide (PI) Fluorescence Assays in the Rat Cortex

**Figure 5:**
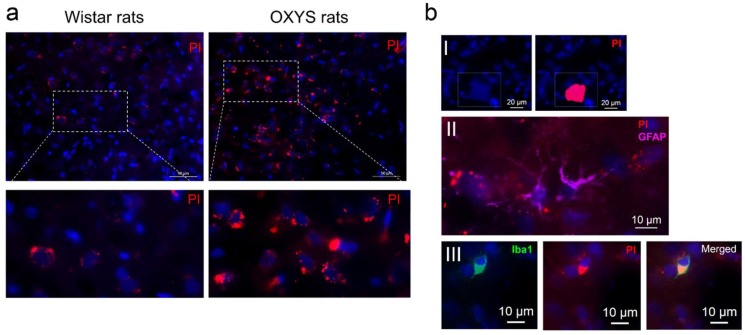
Examples of propidium iodide (PI) fluorescence in the cortex after in vivo administration to the OXYS and Wistar cortex. (**a**) The PI staining dramatically increased in the cortex of old OXYS rats as compared to Wistar rats. The insets depict inclusions of PI in cells. (**b**) Localization of PI+ signals in different cell types: (I) PI staining in nuclei of the cells with necrotic morphology; (II) PI dots in a neuron with viable morphology and no colocalization of PI signals with glial fibrillary acidic protein (GFAP) + astrocytes; (III) Iba1^+^ microglia show PI immunofluorescence in the cytoplasm.

PI staining dramatically increased in the cortex of OXYS rats as compared to Wistar rats. We analyzed colocalization of PI with Iba1 (ionized calcium binding adaptor molecule 1) and glial fibrillary acidic protein (GFAP) in both strains (Figure 5).

We detected PI staining in neurons and some Iba1^+^ microglia, but we detected no colocalization of PI with GFAP + astrocytes (Figure 5bII,III). The PI staining pattern in the cortical cells of Wistar and OXYS rats was different from the commonly expected PI staining pattern [20]. In one case, we observed traditional PI staining in the nuclei of neuron with necrotic morphology (Figure 5bI). In the other case, the strongest PI+ signals were located in cytoplasmic granules and nucleoli of some neurons and microglia (Figure 5b).

## 3. Discussion

Regulated death of cells plays key roles in development and adult-tissue homeostasis. Mounting evidence points to the importance of impairments of apoptosis, autophagy, and necrosis in the pathogenesis of aging and neurodegenerative diseases, especially AD. Our results indicate that in OXYS rats, dysfunction of cell death makes a significant contribution to the development of the AD-like pathology. According to classification of the different types of cell death suggested by European society of cell death there are twelve major cell death paths: Intrinsic apoptosis, extrinsic apoptosis, mitochondrial permeability transition (MPT)-driven necrosis, necroptosis, ferroptosis, pyroptosis, parthanatos, entotic cell death, NETotic cell death, lysosome-dependent cell death, autophagy-dependent cell death, and immunogenic cell death [21]. Here, we studied apoptosis without separation to intrinsic and extrinsic types, autophagy-dependent cell death and necroptosis. We emphasize that we observed regulated forms of necrosis. Necroptosis was examined using RIPKs, PI detected cells with a necrotic structure, which most likely includes necroptosis, ferroptosis, pyroptosis and MPT-driven necrosis and late apoptosis cells.

Autophagy is an important and conserved lysosomal degradation cascade that controls cytoplasm quality by eliminating intracellular aggregated proteins as well as damaged organelles. Which key role is played by autophagy in AD development is still debated. One viewpoint suggests that aberrant autophagy induction leads to concentration of autophagic vacuoles that are rich in APP, Aβ peptide, and the components necessary for its formation; another hypothesis postulates that dysfunctional autophagic clearance or diminution of autophagic efficacy takes part in AD progression [22]. Our results showed disturbances in the autophagy process in the cortex of OXYS rats: Its suppression at late stages of the disease. In the cerebrum, neurons having complex dendritic and axonal structures depend on the efficient proteostasis and active transport to accommodate a dynamic microenvironment in the course of brain aging and development. In this study, we quantified only neuronal bodies containing LC3 A/B proteins. We observed increased numbers of LC3 A/B puncta in OXYS and Wistar rats during aging and the increased numbers of LC3 A/B^+^ neurons in 18-month-old OXYS rats as compared to age-matched Wistar rats. This phenomenon can be explained as follows. The autophagosome accumulation could be caused by either decreased lysosomal clearance or increased induction of autophagy or a combination of the two. We observed a decreased protein level of LC3 A/B and no differences in ATG7 and complex ATG5–ATG12 protein levels in OXYS rats with disease progression. LC3 A/B localizes to the elongated isolation membrane at a later step of autophagosome formation, while the ATG5–ATG12–ATG16L complex dissociates from it. The conjugation of ATG5 with ATG12 is catalyzed by ATG7 and ATG10 [23]. These findings may reflect impairment of the mechanisms regulating the late stage of autophagosome formation during disease progression. Moreover, brain sections of patients with AD feature a build-up of dysfunctional late autophagosomes and autolysosomes [24,25].

It is likely that the final stage of the autophagic–lysosomal degradative pathway is commonly blocked in neurodegenerative diseases [26]. As for AD, the defects in lysosomal acidification contribute to the failure of autophagy-mediated protein degradation [26,27]. Lysosomal proteolysis dysfunction can slow the axonal transport of autophagic compartments and results in an increased number of immature autophagosomes [28]. Indeed, we noticed that ubiquitinated proteins’ levels were significantly higher in the cerebral cortex of 18-month-old OXYS rats than in age-matched Wistar rats. Our previous study suggests that older OXYS rats show accumulation of severely damaged mitochondria [29]. The autophagic disfunction observed in aged OXYS rats is likely due to impaired clearance of autophagic vesicles rather than enhanced autophagosome formation alone [26]. Recently alteration of autophagic flux have been found in the retina of OXYS rats [30]. 

Notably, the observed change in autophagy marker LC3 A/B in the cerebral cortex of the old OXYS rats was accompanied here by an increase in both the number of apoptotic cells and colocalization of necroptosis markers. There is growing evidence suggesting that autophagy likely plays a causative role in neuronal death [26]. Alteration of autophagy may predispose the cell to death. Nevertheless, the exact role of activated autophagy in neuronal death remains controversial, because it is very difficult to determine whether neurons die as a direct consequence of autophagic activation or whether activated autophagy is merely an attempt to promote neuronal survival [26].

Apoptosis and autophagy are important physiological processes contributing to the maintenance of cellular homeostasis in neurons [31]. In a recent study, Ułamek-Kozioł et al. presented the time course of expression of autophagy, mitophagy, and apoptotic gene levels in the medial temporal lobe cortex subjected to transient ischemia [5]. Their data indicate that an efficient autophagic response in the temporal cortex might protect cortical neurons from acute cell death. We found that the numbers of apoptotic cells and neurons were greater in OXYS rats than in Wistar rats at all ages. Wherein we observed a decreased number of apoptotic total cells and TUNEL + NeuN + neurons at age five months in the cortex of both rat strains. This phenomenon could be associated with the resistance of adult mature CNS neurons to cell death compared to postnatal period of brain development [32]. The adult postmitotic neurons have lower sensitivity to the intrinsic pathways of apoptosis to ensure the long-term survival of mature neurons for entire life [8,32]. However, under pathological conditions, extrinsic cell death pathways may be activated and can overcome the resistance of the adult postmitotic neurons to apoptosis, thus promoting both extrinsic apoptosis and necroptosis [8,32]. To the age 18 months both the quantity of apoptotic total cells and RIPK1-and-RIPK3 colocalization were increased in the OXYS cortex. This result is consistent with a recent study showing that necroptosis signaling is dramatically enhanced in AD patients as the protein levels of the RIPK1–RIPK3–MLKL axis increase [2,33].

The natural decline of proteostasis with ageing may contribute to the onset of neurodegeneration by reducing protein turnover, promoting the accumulation of misfolded proteins, and enhancing RIPK1 activation. This age-dependent decline of proteostasis may involve a decrease in the stability of correctly folded proteins as well as deficits in protein turnover via autophagy and the proteasome [8]. Necroptosis can be experimentally activated under apoptosis-deficient conditions [34] and elicits a strong inflammatory response that can drastically alter the local tissue environment and drive the pathogenesis of neurodegenerative diseases [35,36]. RIPK1 kinase-dependent necroptosis serves as a major executioner of cell death in response to extracellular inflammatory signaling [34]. Moreover, RIPK1 has a well-recognized role in the transcription of neuroinflammatory genes in microglia [37].

PI staining is a popular method for detecting cell death [38,39]. This in vivo technique based on the impermeability of an intact cell membrane to PI molecule. Accordingly, PI staining more specific for necrosis detection, however in the case of late apoptosis, the cells may be propidium iodide positive [39]. Expectedly, we observed increased PI staining in the cortex of old OXYS rats as compared to Wistar rats. Nevertheless, PI+ neurons with necrotic morphology were rare in the cortex of both rat strains. Most PI+ cells (neurons and microglia) were characterized by signals in cytoplasmic granules and nucleoli. These PI+ neurons and microglia had the morphology of viable cells. The occurrence of these “false” PI+ neurons and microglia could be attributed to high concentrations of the dye. Despite being regarded as impermeable, cells that are viable can be stained by diffusion-driven uptake if the gradient of concentration at the outer cell wall border is steep enough [40]. We suggest that the increased number of PI+ neurons and microglia in OXYS rats is due to leakiness of the blood–brain barrier and impairment of membrane integrity in old OXYS rats in comparison with Wistar rats. Previously, substantial cerebrovascular anomalies have been noted in the hippocampus of OXYS rats at the age of 18 months, when the cognitive deficits and cerebral neurodegenerative processes are well pronounced [41,42]. Possibly, our results mean that PI can serve as an in vivo marker of an impairment of blood–brain barrier integrity.

**Figure 6:**
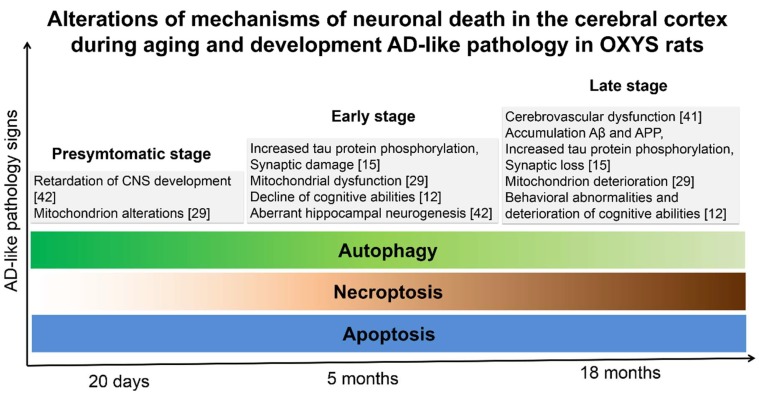
The proposed model of alterations of neuronal cell death mechanisms during the development of the AD-like pathology in OXYS rats. Apoptosis is activated at the pre-symptomatic stage (age 20 days). Progression of the AD-like pathology is accompanied by activation of apoptosis and necroptosis resulting from a decline of proteostasis and of autophagy. Greater color saturation means enhancement of a process.

Taken together, our findings point to the activation of apoptosis and necroptosis at the late stage of the AD-like pathology as a result of a decline of proteostasis and of autophagy (Figure 6).

## 4. Materials and Methods

### 4.1. Animals

All the experimental methods complied with the European Communities Council Directive of 24 November, 1986 (86/609/EEC). The animal experiments were approved by the Bioethics Commission 9 of the Institute of Cytology and Genetics, Siberian Branch of Russian Academy of Sciences (Novosibirsk, Russia) according to The Guidelines for Manipulations with Experimental Animals (the decree of the Presidium of the Russian Academy of Sciences No. 12000-496 of April 2, 1980). Senescence-accelerated OXYS male rats at preclinical (age 20 days, *n* = 10), early (five months, *n* = 10), and late (18 months, *n* = 10) stages of the AD-like pathology as well as male Wistar rats (age-matched controls, *n* = 10 at each age group) were acquired from the Breeding Experimental Animal Laboratory of the ICG, SB RAS. For immunohistochemical analysis, four rats were selected randomly from every age group. Three tissue slices (at least) were studied per animal. For western blot analysis, six rats were chosen randomly from every age group. The body weights of male rats were following: 45.58 ± 1.59 g and 45.33 ± 1.46 g at the age of 20 days (Wistar and OXYS rats, respectively), 356 ± 5 g for Wistar rats and 287 ± 7 g for OXYS rats at the age of five months, 677 ± 22.7 g and 442 ± 8.3 g at the age of 18 months (Wistar and OXYS rats, respectively).

At four weeks of age, the pups were weaned, housed in groups of five per 57 × 36 × 20 cm cage, and maintained in standard laboratory conditions (22 ± 2 °C, 12/12 h light/dark cycle with lights on at 9:00 am, 60% relative humidity). The rats had *ad libitum* access to standard rodent feed (PK-120-1, Ltd., Laboratorsnab, Russia) and water. These experiments were carried out at the Institute of Cytology and Genetics, Siberian Branch of Russian Academy of Sciences (Center for Genetic Resources of Laboratory Animals; RFMEFI61914X0010 and RFMEFI61914X0005).

The OXYS rat strain was developed at the Institute of Cytology and Genetics, Siberian Branch of Russian Academy of Sciences (Novosibirsk), from Wistar rats via selection and inbreeding of highly susceptible rats to cataractogenic effect of a galactose-rich diet. Only five generations of rats were feeded by galactose-rich diet, the subsequent generations of rats developed cataracts spontaneously. The OXYS rats were registered in the Rat Genome Database (http://rgd.mcw.edu/). In addition to spontaneous cataract, the OXYS rats develop accelerated senescence syndrome, which is characterized by early development of thymus involution, retinopathy similar to human age-related macular degeneration (AMD), high blood pressure, senile osteoporosis, and accelerated brain aging with signs of AD [10,12]. Behavioral alterations and learning and memory deficits in OXYS rats develop by the age of 3–5 months against the background of first signs of neurodegeneration: Death of neurons, disruption of synapses, mitochondrial dysfunction, and hyperphosphorylation of the tau protein in the hippocampus and cerebral cortex. With age, neurodegenerative changes in OXYS rats increase, accompanied by overproduction of APP and accumulation of β-amyloid by age 12 months and pronounced amyloid pathology by 16–18 months of age [12,15].

### 4.2. Immunohistochemistry

Sagittal sections of the brain (20 μm thick) from 20-day-old and five- and 18-month-old Wistar and OXYS rats (*n* = four animals per group) were prepared on a Microm HM-505 N cryostat at −20 °C (Microm, Germany) and then put on Polysine-glass slides from Menzel-Glaser (Braunschweig, Germany). After serial washes with PBS, the tissue sections were incubated for 15 min in PBST (PBS containing 0.1% of Triton X-100) and next in 5% bovine serum albumin (BSA; cat. # A3294, Sigma-Aldrich, St. Louis, MO, USA) in PBST at RT for 1 h for blockage of nonspecific binding sites and for permeabilization of the tissues, and then were probed with primary antibodies at 4 °C overnight. The primary antibodies were all diluted in 5% BSA in PBST: These were antibodies to LC3 A/B, NeuN, ubiquitin, RIPK1, and RIPK3 (cat. ## ab128025, ab104224, ab7780, ab72139, and ab62344, respectively, Abcam, Cambridge, MA, USA). After several PBST washes, the sections were incubated with secondary antibodies chemically coupled with Alexa Fluor 488 or 568 (cat. ## ab150073 and ab175472, Abcam) for 1 h at room temperature (RT) and were then washed with PBST. The tissue sections were coverslipped with the Fluoroshield mounting medium supplemented with DAPI (cat. # ab104139, Abcam). Negative controls were processed identically but a primary antibody was not added. The staining signals were counted under a microscope with a 40× objective lens (Axioskop 2 plus, Zeiss, Germany) and then averaged in each group of three slices per animal. The countings were made in a blind or blinded experiment; the brain section group names were withheld (masked or blinded) from observer until after the countings were complete. 

### 4.3. Double Staining (TUNEL and NeuN)

Neural apoptosis was assessed by TUNEL staining via a DeadEnd Fluorometric TUNEL System (cat. # G3250, Promega Bio Sciences LLC, San Luis Obispo, CA, USA) The slides were incubated overnight with the rabbit anti-NeuN antibody (1:200, cat. # ab177487, Abcam). After probing with a relevant secondary antibody (1:200, cat. # ab175470, Abcam), a standard TUNEL procedure was performed as described previously [17]. Nuclei of cells were stained using 4′,6-diamidino-2-phenylindole (DAPI).

### 4.4. The In Vivo PI-Labeling Protocol

PI is a nucleic-acid stain. After the dye binds to nucleic acids, the fluorescence of this dye increases 20–30-fold. Given that PI cannot cross the plasma membrane, it is also employed to detect necrotic cells [43]. So after the plasma membrane is disrupted, PI gets into the cell and intercalates into RNA and DNA. In late apoptotic and necrotic cells, the integrity of the plasma and nuclear membranes decrease. In late apoptotic and necrotic cells, the integrity of the plasma and nuclear membranes decrease. Thus, PI is more specific marker for detection of necrotic cells but late apoptosis cells also may be PI-positive [39].

PI (30 µg/mL, 3 mg/kg, Sigma) was injected intraperitoneally 2 h before euthanasia into 16-month-old OXYS (*n* = 5) and Wistar rats (*n* = 5). The group of 16-month-old rats refer to old animals. Earlier, we examined in detail the dynamics of AD-like pathology development in OXYS rats and found that the increased level of b-amyloid and tau protein is established to the age of 12 months [12]. Since we proposed that the age of 16 months corresponds to the stage of active disease progression and does not differ in their severity of AD signs from the age of 18 month [15]. The brains were collected and fixed for 48 h in fresh 4% paraformaldehyde/PBS with subsequent cryoprotection in 30% sucrose/PBS for 48 h at 4 °C; after that, the brains were frozen and stored at –70 °C before processing. The slices (20 μm thick) prepared on the cryostat were transferred to Polysine^®^ glass slides (Menzel-Glaser) that were cover-slipped with the Fluoroshield mounting medium supplemented with DAPI, and were inspected under the fluorescence microscope Axioplan 2 (Zeiss). For PI, excitation/emission filters at 568/585 nm were chosen.

#### PI Labeling and Multimodal Immunohistochemistry

For immunohistochemical detection of PI-labeled neurons, astrocytes and microglia, the brain sections prepared on the cryostat were incubated for 15 min in PBST and in 5% BSA (cat. # A3294, Sigma Aldrich) in PBST for 1 h at RT to block nonspecific binding sites and to permeabilize the tissues, and then were incubated overnight with primary antibodies at 4 °C. The sections were incubated with the rabbit anti-GFAP antibody (cat. # ab7260, Abcam) to label astrocytes, and the goat anti-Iba1 antibody (cat. # ab5076, Abcam) to label microglia at 1:100 dilution in 5% BSA. Next, the tissue sections were washed briefly in PBS and were then incubated with secondary antibodies conjugated with Alexa Fluor 488 or 647 (cat. ## ab150113 and ab96886, Abcam) at RT for 1 h and were then washed in PBST. Cell nuclei were stained with DAPI (cat. # ab104139, Abcam).

### 4.5. Western Blots

Immunoblotting was carried out as described previously [16]. The following antibodies were employed in this study: Anti-ATG7 (1:2000 dilution), anti-ATG12 (1:1000), LC3 A/B (1:500), and anti-β-actin (1:2000) (cat. ## ab133528, ab155589, ab128025, and ab6276, respectively, Abcam). After blocking in 5% BSA/PBS containing 0.1% of Tween 20 for 1 h, these membranes were probed with the primary antibodies overnight at 4 °C. After incubation with a secondary antibody (1:3000), chemiluminescent signals were registered and scanned, and the band intensity was quantified using ImageJ software. β-actin was used always as the internal loading control.

### 4.6. Statistics

Data were assessed by analysis of variance (ANOVA in software Statistica 8.0). The *post hoc* test was performed on significant main effects and their interactions to evaluate differences between some groups of means. The results are shown as mean ± SD. Differences were regarded as statistically significant at *p* < 0.05.

## 5. Conclusions

Finally, we propose that defective or inadequate autophagy supports neuronal death, e.g., apoptosis and necrosis, in the OXYS strain, which spontaneously develops brain alterations resembling AD (Figure 6). Our results are consistent with the idea that the nature of alterations in the pathways of apoptosis, autophagy, and necroptosis depends on the stage of the AD development.

## Abbreviations

AD	Alzheimer’s disease
APP Aβ CNS DRs	Amyloid precursor protein β-amyloid peptide Central nervous system Death receptors
TUNEL	Terminal deoxynucleotidyl transferase dUTP nick end labeling
RIPK PI MLKL	Receptor-interacting protein kinase Propidium iodide Mixed-lineage kinase domain-like protein
